# Potential biomarkers of atopic dermatitis

**DOI:** 10.3389/fmed.2022.1028694

**Published:** 2022-11-17

**Authors:** Ling Yu, Linfeng Li

**Affiliations:** ^1^Department of Dermatology, Beijing Friendship Hospital, Capital Medical University, Beijing, China; ^2^Department of Dermatology, Henan Provincial People’s Hospital, Zhengzhou, China; ^3^Department of Dermatology, Zhengzhou University People’s Hospital, Zhengzhou, China

**Keywords:** atopic dermatitis, biomarker, predictor, phenotype, precise treatment

## Abstract

Atopic dermatitis (AD) is a chronic, recurrent inflammatory skin disease with a wide range of heterogeneity. Accurate biomarkers or predictors are the keys to instructing personalized tailored precise treatment. The development of technology such as transcriptomics, genomics, and proteomics provides novel insights into the possibility to find potential biomarkers. Meanwhile, emerging minimally invasive methods such as tape stripping were used to reveal different profiles of patients’ skin without biopsy. Several potential biomarkers or predictors have been found. In this review, we summarized the current development of potential biomarkers of AD. Nitric oxide synthase 2/inducible nitric oxide synthase (NOS2/iNOS), human beta-defensin-2 (hBD-2), and matrix metalloproteinases 8/9 (MMP8/9) may be the candidate biomarkers for AD diagnosis. Filaggrin (FLG) gene mutation increased the occurrence risk of AD. Fatty-acid-binding protein 5 (FABP5) may serve as an effective biomarker for the atopic march (AM). Squamous cell carcinoma antigen 2 (SCCA2), serum thymus and activation-regulated chemokine (TARC), cutaneous T-cell-attracting chemokine (CTACK), eosinophil-derived neurotoxin (EDN), macrophage-derived chemokine (MDC), lactate dehydrogenase (LDH), and interleukin (IL)-18 can be the candidate biomarkers for disease severity monitoring. IL-17, IL-23, IL-33, and indoleamine 2,3-dioxygenase (IDO1) can be used as predictive biomarkers for AD comorbidities. LDH, TARC, pulmonary and activation-regulated chemokine (PARC), periostin, IL-22, eotaxin-1/3, and IL-8 may be the candidate biomarkers for monitoring treatment effects. There are still unmet needs and a long way to go for more convenient, non-invasive, and effective predictors and biomarkers to better guide personalized precise treatment.

## Introduction

Atopic dermatitis (AD) is a common chronic, recurrent inflammatory skin disease that is characterized by acute eczematous or chronic lichenified lesions with a wide range of heterogeneity. It affects almost 10–20% of people around the world (1). The intense itching, accompanied by psychological pressure and economic burden, greatly affects people’s quality of life (2). The mechanism of AD occurrence is multifactorial, genetic factor of filaggrin (FLG) gene mutation, epidermal barrier dysfunction with decreased diversity of the microbiome, environmental factors of allergens and irritants permeation, type 2 skin inflammation activation, immunological dysregulation, and neuroimmune induced itch-scratch cycle, all of these factors lead to the occurrence of AD (3). Patients with AD always appear to be associated with atopic diathesis, accompanied by personal or family history of atopy comorbidities. Their condition may have heterogeneous trajectories (4), some people are transient, some relapse, while others progress to persistent and develop allergic rhinitis, asthma, or allergic conjunctivitis. Various diagnostic criteria (5–7) and different typical or atypical morphologies and distributions also make AD a complex disease. Furthermore, predictors or biomarkers of AD that can be used to screen and diagnose AD precisely are still lacking.

Patients with AD can be stratified according to different conditions. Since clinical manifestation is age related, it can be divided based on age. It can also be stratified based on the onset age and the natural course of AD, including the very early onset (between 3 months and 2 years) with or without remission, early onset (between 2 and 6 years), childhood-onset (between 6 and 14 years), adolescent-onset (between 14 and 18 years), adult-onset (between 20 and 60 years), and very late-onset (>60 years) (8). Furthermore, the endophenotype combining clinical phenotype with genotype is also suggested to evaluate the biological nature of the disease. There are also differences between different ethnicities and races (9). Although many heterogeneities of different clinical manifestations, phenotypes, atopic march (AM), and prognoses have been found (10, 11), AD is still considered one disease that is treated with almost the same strategy. Recently, new targeted biologicals such as dupilumab (12) and small-molecule drugs such as Janus kinase (JAK) inhibitors (13) have been approved to treat AD. More accurate phenotype biomarkers or predictors will be the key to instructing personalized tailored precise treatment.

Nowadays, the development of technology such as transcriptomics, genomics, proteomics, and deep next-generation sequencing can be used as tools for differential analysis (14–17), which provides the possibility to find potential biomarkers. At the same time, minimally invasive methods such as tape stripping (18–21) were used to examine various components of patients’ skin without biopsy. All the development contributes to exploring more valuable biomarkers or predictors to help disease diagnosis, classification, disease severity monitoring, and therapeutic effect and prognosis prediction. Herein, we introduce the current development of biomarkers or predictors for AD.

## Biomarkers for atopic dermatitis diagnosis

Different diagnostic criteria, which are mainly based on clinical symptoms and manifestations, personal or family atopic diathesis, and some may include relevant laboratory tests (such as blood eosinophils, serum immunoglobulin E [IgE], and allergen tests), are used for the diagnosis of AD. There are unmet needs for specific indicators to confirm the diagnosis and distinguish AD from other diseases with similar manifestations.

For the differentiation of AD and psoriasis, studies have shown that the expression of interleukin (IL)-36γ (IL-1F9) in the skin lesions of patients with psoriasis is significantly increased, which can be used to distinguish between AD and psoriasis (22). However, the expression levels of both serum IL-36γ and skin IL-36γ increased in AD and mycosis fungoides/Sézary syndrome, and no statistically significant differences were found, which means it is not a perfect biomarker for AD diagnosis (23).

In addition, the expression levels of human beta-defensin-2 (hBD-2) in the skin and serum of patients with psoriasis increase, and its expression levels are closely related to the Psoriasis Area and Severity Index (PASI) score. The expression level of hBD-2 in psoriasis is significantly higher than in patients with AD. Thus, hBD-2 can be used to differentiate psoriasis from AD (24). Transcriptome analysis was also conducted by tape stripping, which showed that the RNA profile of skin tape stripping from non-lesional skin of patients with AD was more similar to that of lesional skin. AD showed T-helper (Th) 2 skewing in both lesional skin and non-lesional skin, with a significant increase in Th2-related factors (IL-4R, IL-13, C-C motif chemokine ligand [CCL]17/serum thymus and activation-regulated chemokine [TARC], CCL24/eotaxin-2), while, the pruritus mediator IL-31 expressed only increase in lesional skin of patients with AD. Psoriasis expressed preferential Th17 skewing, with a significant increase in Th17-related factors (IL-17A/F and IL-36A/G). The Th1-related cytokines (interferon [IFN]-γ and C-X-C motif chemokine ligand [CXCL]9/CXCL10) were also upregulated. No significant differences were found in the Th22-related products in both AD and psoriasis; the epidermal barrier-related terminal differentiation, tight junctions, lipid biosynthesis, and metabolism markers were all downregulated in these two diseases. Although levels of inducible co-stimulator (ICOS) (T cell activation marker) and CD1a (expressed by DC and Langerhans cells) increased and PSORS1C2 (terminal differentiation) and CDH12 (cadherin marker) decreased only in AD, the quantification of innate immunity-related marker nitric oxide synthase 2/inducible nitric oxide synthase (NOS2/iNOS) in the tape strips of lesional skin by quantitative PCR was identified to be a potential good biomarker to differentiate AD and psoriasis accurately (19). Furthermore, the immunofluorescence stain of NOS2/iNOS in the paraffin-embedded pathological section of AD and psoriasis showed that the expression of NOS2/iNOS was more prominent in psoriasis. In addition, the different expression locations of CCL27 (expressed in the nucleus of eczema and the cytoplasm of psoriasis lesion) could distinguish eczema from psoriasis (25).

In addition, the carbonic anhydrase II (CA II) gene is highly expressed in the epidermis of all forms of eczema, including AD, allergic contact dermatitis (ACD), and irritant contact dermatitis, but not in psoriasis. Chemokines CXCL10 and CCL17 express high levels in the epidermis of ACD. Meanwhile, the neuron-specific Nel-like protein 2 (NELL 2) is highly expressed only in the epidermis of AD, not in psoriasis (26). It may be associated with the nerve fibers in the lesional skin, which can better distinguish AD from other diseases (27).

A skin surface saline wash sampling method was used to detect matrix metalloproteinases (MMP) in the lesional skin of patients with AD. It found that the activity of MMP, especially MMP-8 and MMP-9, increased 10–24-fold in the skin lesions of patients with AD when compared with normal controls and increased 5-fold when compared with unaffected skin of patients with AD (28).

About AD differential diagnosis, major studies have focused on distinguishing AD from psoriasis, however, studies on the expression of the potential biomarkers in other diseases that can mimic AD are insufficient. The NOS2/iNOS, hBD-2, and MMP8/9 may be the candidate biomarkers for AD diagnosis. There was also a study exploring the urinary lipid profile in patients with AD and found increased levels of prostaglandins metabolites and arachidonic acid metabolite, which may help to find novel urinary biomarkers for AD diagnosis (29).

## Biomarkers for atopic dermatitis occurrence and progression

The heterogeneities of AD determine the different potential occurrences and progression of different allergic diseases in patients with AD. There is a consensus that allergic diseases exist in AM in a time-based order, which is defined as individuals evolving from AD and food allergy to allergic rhinitis, asthma, and other typical atopic diseases. However, not all the atopic marches progress in a fixed pattern completely (30). Meanwhile, predictors for the potential progression to other allergic diseases are still lacking.

Air pollution and environmental exposure to CO, NO_2_, NO, and O_3_ during prenatal and early life are important factors that could affect the development of eczema, asthma, and other allergic diseases in children (31, 32). Higher prenatal CO exposure had a higher risk of AD development in children before 6 months (31). Prenatal exposure to NO_2_ and its changes in concentration over time were predictors of adolescent AD and allergic rhinitis (33).

The study also suggested that preterm infants had a lower risk of AD than full-term infants. Schoch et al. (34) found that the time in the intensive care unit was statistically significantly correlated with the low incidence of AD in premature infants. Another study showed that premature babies were at increased risk of asthma, while, overdue delivery was associated with AD (35). Maternal allergic status is also associated with an increase of IL-4(+) CD4(+) T cells and a decrease in the Treg/Th2 ratio in umbilical cord blood at birth, which increases the risk of developing AD (36). The risk of AD is also increased in neonatal with adiposity during the first year of life (37).

Dermatitis at birth ≤3 months was significantly associated with sensitization, AD, and food allergy. Almost all infants with food allergies experience dermatitis at ≤3 months of age. In infants with dermatitis ≤3 months, breastfeeding was significantly associated with sensitization and food allergy (38). Ochiai et al. compared the levels of related cytokines in breast milk and found that a high concentration of eotaxin in mature breast milk (collected at 1 month postpartum) is a risk factor for AD at 6 months of birth (39).

More than half of patients with AD have food allergies, and 44.9% of patients with AD have high eosinophil levels. The incidence of food allergy was almost 70.8% in the high-level eosinophil group. Therefore, high-level eosinophil means a high risk of food allergy in patients with AD (40).

Dry skin can also be used as a predictor of AD (41). Transepidermal water loss (TEWL) of infants at 2 days and 2 months of age can be a strong predictor of AD at 12 months (42). The level of trihydroxy-linoleic acid is related to the TEWL of the forearm of AD patients without skin lesions, detecting the level of trihydroxy-linoleic acid in epidermal cuticle by tape-stripping can serve as a potential biomarker to reflect the skin barrier function of patients with AD (43). Filaggrin gene deletion mutation is an important risk factor for AD (44), different genotypes were studied as well, especially the FLG P478S GG genotype significantly increased the risk of AD. In addition, the GG genotype also significantly increased the risk of asthma and allergic rhinitis in patients with AD (45). However, studies also pointed out that increased TEWL in neonates is a strong predictor of AD development and food allergy regardless of the status of the filaggrin gene (46). Four lipid markers, especially the phytosphingosine level at 2 months of age in children who developed AD were lower than those who did not, which may predict the occurrence of AD. Meanwhile, TARC/CCL17 levels were higher in those who developed AD than in the normal control (47).

As for the development and progression of AD, allergic family history is a strong predictor of developing multiple other allergic diseases from adolescence to adulthood (48). At the same time, food allergy in the first 2 years of life (with or without air-borne allergens) increases the risk of subsequent asthma and allergic rhinitis (49).

Fatty-acid-binding protein 5 (FABP5) in the skin and T cells of patients with AM and AM murine models were all positively related to IL-17A levels in the skin and serum. It pointed out that FABP5 can be involved in the development and progression of the atopic process by promoting Th17 inflammation, and FABP5 may serve as an effective biomarker for atopic march (50).

## Biomarkers for monitoring atopic dermatitis severity

Toll-like receptor 2 genes (TLR2)-16934A > T polymorphism can affect its transcriptional activity and is associated with Scoring Atopic Dermatitis (SCORAD), which makes it to be a predictor of AD severity (51).

The imbalance of skin microflora or the abundance of *Staphylococcus aureus* was related to the severity of AD, high baseline *S. aureus* abundance could predict AD severity after 8 weeks (52). However, many pitfalls need to be overcome, lacking of standardized microflora sampling and skin microflora detecting methods tends to cause information bias, and further research norms are still needed to make skin *S. aureus* a better clinical biomarker (16).

Serum TARC/CCL17, an important chemoattractant of T cells, is considered a biomarker for monitoring the severity of AD in adults in daily practice (53). Serum eosinophil-derived neurotoxin (EDN) is associated with the severity of AD and can also predict the relapse of severe refractory AD, which can be used as a candidate biomarker for predicting the severity of disease (54). Squamous cell carcinoma antigen 2 (SCCA 2), also known as serine protease inhibitor B4 antibody (SERPINB4), is correlated with SCORAD score and decreases significantly with the improvement of the disease. It is believed to be a reliable biomarker of AD severity in both adults and children. TARC varies significantly among patients of different ages, nevertheless, age has little influence on SCCA2, which is more convenient for clinical application, so SCCA2 is considered superior to TARC and IgE in the severity assessment of AD in children (55, 56). Although serum SCCA2 levels increase both in patients with AD and psoriasis compared with healthy people, the serum SCCA2 level of AD is significantly above psoriasis, which is comparable (55). The concentrations of microbicidal peptide human neutrophil α-defensins, dermcidin and Th2-related chemokines CCL17, macrophage-derived chemokine (MDC)/CCL22, and cutaneous T-cell-attracting chemokine (CTACK)/CCL27 in patients with AD were significantly increased, among them CCL27 and CCL22 were positively correlated with SCORAD, meanwhile, CTACK was positively correlated with pruritus in patients with AD (57). Serum IgE had been studied as a biomarker, however, the meta-analysis revealed only a moderate correlation to disease severity, which meant that it was not the ideal biomarker. The serum lactate dehydrogenase (LDH), serum E-selectin (SELE), serum IL-18, and serum eosinophil cationic protein were also studied, and results showed that serum LDH and IL-18 could be better potential candidate biomarkers for AD severity (58).

The emerging minimal invasive method of tape stripping contributes to revealing the transcriptomic profile of patients with AD, which provides novel insights into the possibility to find potential biomarkers (21, 59). The global transcriptomic profiling of tape strips obtained from lesional skin and non-lesional skin of patients with AD was detected and analyzed. Many genes were differentially expressed. Non-lesional AD skin tape strip transcriptomes seem to better assay the stratum corneum than skin biopsy transcriptomes, the clinical severity is strongly related to the type 2-high gene signature in AD non-lesional skin (60). AD-preferred Th2 skewing, TARC, and CTACK levels were significantly correlated with the severity of AD in both skin lesions and non-skin lesions in children with AD using the tape stripping method and so did IL-8, IL-18, and Vascular Endothelial Growth Factor (VEGF), among these three cytokines, IL-8 showed the highest correlation with the disease severity in patients with AD (61–63). While no significant correlation was found in skin biopsy specimens (61). The genes of the epidermal barrier and junction (FLG, FLG2, and CLDN8), lipid synthesis and metabolism (FA2H and ELOVL3), and negative immune regulators (IL-34 and IL-37) were downregulated in the lesional skin of patients with AD (19). The barrier-related mRNAs and TEWL were negatively correlated with body surface area (BSA) and pruritus score (64, 65). Genes expression of cellular markers expressed significantly increased, including T cells (CD3), AD-associated dendritic cells (Fc ε RI, and OX40L) and inflammatory markers (MMP12), innate immunity (IL-8 and IL-6), Th2 (IL-4, IL-13, and chemokines CCL17/TARC, CCL26/eotaxin-3), Th17/Th22 (IL-19, IL-22, IL-17F, IL-26, IL-36G, and S100As) and T-regs (FOXP3), which were positively correlated with Total Sign Score and Investigator’s Global Assessment (IGA) Score (65, 66). Levels of IL-1β, IL-18, and thymic stromal lymphopoietin (TSLP) positively correlated with SCORAD and TEWL, while, IL-1α negatively correlated with SCORAD and TEWL. CCL17, CCL22, TSLP, IL-22, and IL-17A also correlated with TEWL (20, 67).

Proteomic analysis of AD also identified cluster proteins correlated with the abnormal skin barrier, especially the SERPINB3, gelsolin (GSN), and KRT77. These proteins were positively correlated with TEWL, serum IgE, and allergic sensitization to food and aeroallergens (68).

## Biomarkers for comorbidities in patients with atopic dermatitis

Atopic dermatitis is regarded as a systemic disease currently. Patients with AD may have allergic rhinitis, asthma, allergic conjunctivitis, and other allergic comorbidities (69). In addition, studies have shown that chronic systemic inflammation contributes to other comorbidities in patients with AD, such as infection, neuropsychiatric, autoimmune, metabolic diseases, or even cardiovascular diseases (70).

The polymorphism of the toll-like receptor 2 genes (TLR2-16934A > T) in patients with AD with total IgE ≥ 106 IU/ml is associated with asthma, allergic conjunctivitis, or atopic family history. TLR2-16934 A > T polymorphism may be a genetic predictor of the coexistence of asthma, atopic conjunctivitis, and family history of atopic disease in patients with AD, especially in subjects with higher IgE (51).

The Th17 pathway and the IL-17 cytokine family may be involved in the development of allergic inflammation, and levels of serum IL-17 correlated with disease severity in patients with allergic rhinitis (71). Meanwhile, the Th17/IL-23 pathway is also involved in the occurrence of asthma, and the serum IL-23 level in asthmatic children is significantly higher than that in healthy controls. Serum IL-23 can be used as an indicator of bronchial function impairment in children with allergic asthma (72).

Serum biomarker profiles were also studied, and the results showed that inflammatory biomarkers increased, especially the levels of IL-5, IL-1β, IL-7, IL-1R1, and IL-15 in the serum of patients with AD, which also supported the idea that AD was a systemic disease (73). In addition, ST2/IL-33 axis regulates Th2 and Th17 immune response in AD and allergic airway disease, which is also associated with cardiovascular diseases (74, 75).

In AD patients with acute eczema herpeticum eruption, serum tryptophan activity significantly decreased, and the expression and activity of indoleamine 2,3-dioxygenase (IDO1) in Langerhans cells isolated from blood also increased. IDO1 seems to be a predictive biomarker for the risk of developing eczema herpeticum in patients with AD (76).

## Biomarkers for monitoring treatment effects in patients with atopic dermatitis

To precisely monitor the treatment effect and predict the underlying adverse reactions, potential biomarkers or predictors are needed and have been explored. There were controversies about the correlation between serum IgE and the treatment response, some demonstrated that the dynamic changes in serum IgE levels could reflect the response of dupilumab to AD (77), while others showed that the pooling of data demonstrated a weak correlation between serum IgE and follow-up disease severity after treatment, which indicated that serum IgE was not the best appropriate biomarker of AD (58). LDH can also serve as a potential serological marker to predict the therapeutic effects of dupilumab (78). Serum biomarkers of TARC, pulmonary and activation-regulated chemokine (PARC), periostin, IL-22, and eosinophil-activated chemokines (eotaxin-1 and eotaxin-3) decreased significantly after dupilumab treatment in adult patients with moderate-severe AD (79).

Tape stripping was also applied to search for potential biomarkers to monitor the treatment effect. Dupilumab and topical mometasone treatment can regulate a variety of important immune- and skin barrier-related biomarkers, while immune markers associated with general inflammation (MMP12), Th2 (CCL13, CCL17), Th17/Th22 (IL-12b, CXCL1, S100A12), and innate immunity (IL-6, IL-8, IL-17C) were significantly reduced after dupilumab or topical mometasone treatment (65, 80). Meanwhile, the atherosclerotic/cardiovascular risk proteins, such as SELE/E-selectin, IGFBP7, CHIT1/chitotriosidase-1, and AXL/tyrosine-protein kinase receptor UFO were also suppressed after dupilumab treatment (80). The expression of TARC and IL-8 decreased significantly after treatment with a moisturizer containing ceramide and magnesium in moderate AD, they were also associated with disease severity, which suggested that TARC and IL-8 were potential biomarkers for monitoring disease severity and local treatment effect of AD (18). Another study also identified that the concentration of IL-8 in the stratum corneum decreased significantly after topical corticosteroid treatment, which proved its correlation to the severity of local skin inflammation in patients with AD. Therefore, IL-8 in the stratum corneum can be a potential biomarker to monitor the therapeutic effects (62). Topical corticosteroid treatment resulted in significant reductions in IL-13 and IL-4R mRNA levels in skin biopsies; however, no significant differences in cytokine protein levels were found in tape strips (81).

## Conclusion

**FIGURE 1:**
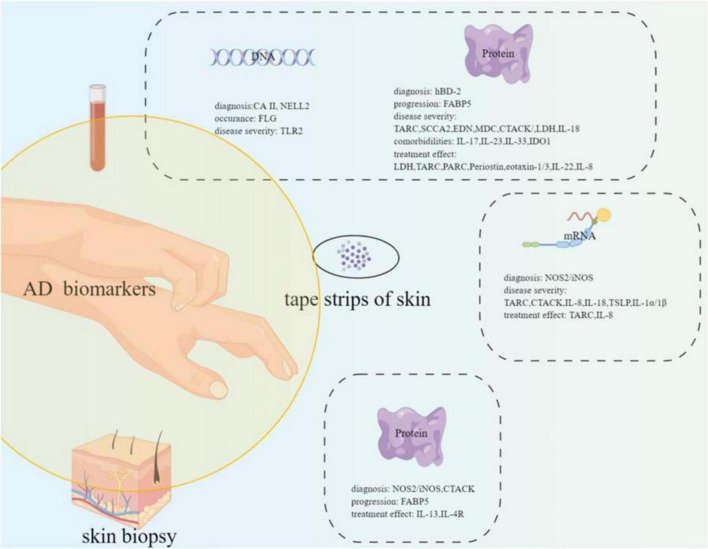
Summary of the potential biomarkers of atopic dermatitis with different samples (blood, tape strips of skin, and skin biopsy) from the patients.

A great deal of effort has been taken to find biomarkers or predictors for AD; however, no perfect biomarkers have been rendered into daily practice yet. Several potential biomarkers or predictors have been suggested. NOS2/iNOS, hBD-2, and MMP8/9 may be the candidate biomarkers for AD diagnosis. FLG gene mutation increased the occurrence risk of AD. FABP5 may serve as an effective biomarker for the atopic march. TARC, SCCA2, CTACK, EDN, MDC, LDH, and IL-18 can be the candidate biomarkers for disease severity monitoring. IL-17, IL-23, IL-33, and IDO1 can be used as predictive biomarkers for AD comorbidities. LDH, TARC, PARC, periostin, IL-22, eotaxin-1/3, and IL-8 can be the candidate biomarkers for monitoring treatment effects (refer to Figure 1, drawn by Figdraw).^1^

## Author contributions

LY and LL researched the data, contributed to the discussion, wrote the manuscript, and reviewed the manuscript. Both authors contributed to the article and approved the submitted version.

## Footnotes

1
http://www.figdraw.com

## Abbreviations

AD: atopic dermatitis
JAK: Janus kinase
FLG: filaggrin
IL: interleukin
TEWL: transepidermal water loss
hBD-2: human beta-defensin-2
PASI: Psoriasis Area and Severity Index
CCL: C-C motif chemokine ligand
CXCL: C-X -C motif chemokine ligand
DC: dendritic cell
NOS2/iNOS: nitric oxide synthase 2/inducible nitric oxide synthase
TARC: serum thymus and activation-regulated chemokine
PARC: pulmonary and activation-regulated chemokine
CTACK: cutaneous T-cell -attracting chemokine
TSLP: thymic stromal lymphopoietin
CA II: carbonic anhydrase II
NELL 2: Nel-like protein 2
ACD: allergic contact dermatitis
IFN: interferon
MMP: matrix metalloproteinases
SCORAD: Scoring Atopic Dermatitis
FABP5: fatty-acid-binding protein 5
AM: atopic march
TLR: toll-like receptor
EDN: eosinophil-derived neurotoxin
SCCA 2: squamous cell carcinoma antigen 2
SERPINB: serine protease inhibitor B antibody
LDH: lactate dehydrogenase
IGA: Investigator’s Global Assessment
IDO1: Indoleamine 2,3-dioxygenase
GSN: gelsolin
ICOS: inducible co-stimulator
SELE: E-selectin.
